# Micromanaging Abdominal Aortic Aneurysms

**DOI:** 10.3390/ijms140714374

**Published:** 2013-07-11

**Authors:** Lars Maegdefessel, Joshua M. Spin, Matti Adam, Uwe Raaz, Ryuji Toh, Futoshi Nakagami, Philip S. Tsao

**Affiliations:** 1 Department of Medicine, Karolinska Institute, Stockholm SE-17176, Sweden; E-Mail: lars.maegdefessel@ki.se; 2 Division of Cardiovascular Medicine, Stanford University, Stanford, CA 94305-5406, USA; E-Mails: josh.spin@gmail.com (J.M.S.); matti.adam@stanford.edu (M.A.); uraaz@stanford.edu (U.R.); rtoh1214@hotmail.com (R.T.); saruwaku@gmail.com (F.N.)

**Keywords:** microRNA, aortic aneurysm, fibrosis, vascular smooth muscle cells, inflammation, biomarker

## Abstract

The contribution of abdominal aortic aneurysm (AAA) disease to human morbidity and mortality has increased in the aging, industrialized world. In response, extraordinary efforts have been launched to determine the molecular and pathophysiological characteristics of the diseased aorta. This work aims to develop novel diagnostic and therapeutic strategies to limit AAA expansion and, ultimately, rupture. Contributions from multiple research groups have uncovered a complex transcriptional and post-transcriptional regulatory milieu, which is believed to be essential for maintaining aortic vascular homeostasis. Recently, novel small noncoding RNAs, called microRNAs, have been identified as important transcriptional and post-transcriptional inhibitors of gene expression. MicroRNAs are thought to “fine tune” the translational output of their target messenger RNAs (mRNAs) by promoting mRNA degradation or inhibiting translation. With the discovery that microRNAs act as powerful regulators in the context of a wide variety of diseases, it is only logical that microRNAs be thoroughly explored as potential therapeutic entities. This current review summarizes interesting findings regarding the intriguing roles and benefits of microRNA expression modulation during AAA initiation and propagation. These studies utilize disease-relevant murine models, as well as human tissue from patients undergoing surgical aortic aneurysm repair. Furthermore, we critically examine future therapeutic strategies with regard to their clinical and translational feasibility.

## 1. Abdominal Aortic Aneurysm Disease

Abdominal aortic aneurysms (AAAs) are defined as permanent dilations of the abdominal aorta. The diagnosis of AAA is commonly an accidental finding, although an increasing number of screening programs target particularly high-risk populations [1]. Screening demonstrates that disease prevalence ranges from 1.3% (45–54 years of age) to 12.5% in men (75–84 years of age), and in women from 0% in the youngest to 5.2% in the oldest age groups [2]. Some recently performed analyses, however, suggest lower prevalence in certain subpopulations [3]. The most feared clinical consequence of AAA progression is acute rupture, which carries a mortality of ~80% [4]. The number of deaths attributed to AAA rupture is around 15,000 annually in the United States [5]. However, this incidence is likely underestimated, since AAA rupture is often not recognized as the cause of death. As many as 60% of patients with AAAs die of other cardiovascular causes, such as myocardial infarction or stroke, thereby suggesting a relationship between AAAs and atherosclerosis [6].

Known predictors of AAA growth include diameter of the aorta at diagnosis and active smoking [7]. Some studies have demonstrated that the incidence and progression of AAA are also related to hypertension and age [8]. However, smoking is considered to be the major modifiable risk factor for development of AAA. Indeed, AAA is more closely associated with cigarette smoking than any other tobacco-related disease, excepting lung cancer. The vast majority of AAA patients (>90%) have a history of smoking [9]. As mentioned above, the prevalence of AAAs is greater in men than in women. However, there is emerging evidence that women present with an increased risk of AAA rupture at smaller aortic diameters than men [10,11].

To date, the only available treatment option for AAA has been surgical repair [1]. The classic approach includes the insertion of an intraluminal graft via open access to the aneurysmal aorta. This has now largely been replaced by endovascular stenting approaches. Besides their lack of indication in early stages of the disease, the current interventional methods carry significant operative risk, and thus appear effective only in preventing aortic rupture [4]. Until now, no pharmacological approach has been identified which effectively limits AAA progression or the risk of rupture in humans. What has been lacking is a detailed understanding of the mechanisms of AAA initiation and expansion.

## 2. Pathology and Cellular Mechanisms

Others have previously discussed the multiple potential cellular and molecular mechanisms associated with AAA development [12,13]. In this article, we will primarily focus on recognized crucial molecular and cellular patho-mechanisms in aneurysm development that are subject to microRNA (miR) regulatory control. Modulation of these miRs could evolve into new therapeutic strategies on the molecular level to combat the burden of aortic aneurysms.

The complex pathology of AAAs is characterized by progressive aortic dilation, promoted by an imbalance between vascular smooth muscle cell (VSMC) proliferation and apoptosis, as well as impairment of extracellular matrix (ECM) synthesis and degradation. These effects are due (at least in part) to transmural aortic inflammation and its disruptive effects on vessel wall homeostasis [8,14,15].

### 2.1. Impaired Homeostasis of Vascular Smooth Muscle Cells and Extracellular Matrix

Inherited syndromes associated with aneurysm formation suggest the importance of disruption of VSMC and ECM homeostasis in aortic dilation [16], although these familial conditions are more typically associated with ascending thoracic aortic aneurysms (TAAs). The aortic pathology of TAA is characterized by elastic fiber fragmentation and loss, proteoglycan accumulation, as well as focal or diffuse regional VSMC degradation and loss [17]. The role of TGF-β signaling dysregulation in this process is complex. Marfan Syndrome (MFS) and Loeys-Dietz Syndrome (LDS), caused respectively by mutations in fibrillin-1 and TGF-β receptors I and II, predispose to ascending thoracic aortic aneurysms (TAAs), but are much less often associated with AAA [18]. The same is true of familial SMAD3 mutations [19,20].

While considerable evidence points to excessive TGF-β signaling in the various familial TAA-associated conditions, animal models have connected AAA to decreased TGF-β activity [21]. While TGF-β receptor 2 is down-regulated in human AAA tissues [22], no association has been found between genetic polymorphisms in TGF-β receptors and serum TGF-β1 concentration in humans with AAA [23]. Systemic blockade of TGF-β activity augments AngII-induced AAAs in C57BL/6 mice as well as hypercholesterolemic mice, and appears associated with VSMC apoptosis, elastin degradation, and increased inflammatory activity in the aortic wall. In a rat model with chimeric aneurysms located in the infrarenal aorta, TGF-β1 overexpression via endovascular delivery of an adenoviral construct stabilizes pre-existing aortic aneurysms [21].

As a side note, TAAs also occur in families (without syndromic features) due to mutations in SMC contractile protein genes, including SMC-specific isoforms of α-actin (ACTA2) and myosin heavy chain (MYH11), along with the kinase that controls SMC contraction (MYLK) [17,24,25].

Multiple matrix metalloproteases (MMPs), which degrade ECM and are important regulators of aortic vessel wall integrity and morphology, have been extensively studied in human AAA, MMP-9 in particular. A recent meta-analysis included eight case-control studies comparing blood MMP-9 concentration between patients with AAAs and control subjects. Despite wide heterogeneity in circulating levels (30–750 ng/L), significantly higher MMP-9 concentrations were found in AAA patients [26].

### 2.2. Inflammation

Various inflammatory cell types are enriched in AAA tissues, especially macrophages. In a rabbit model of AAA induced by periaortic application of calcium chloride, there is striking macrophage accumulation within the adventitia [27]. This feature is also observed in porcine pancreatic elastase (PPE)-infusion induced AAAs in rats [28]. In ApoE^−/−^ mice infused with angiotensin II (AngII), macrophage infiltration within the medial layers of the aorta is accompanied by medial rupture as an early characteristic [29], while profound accumulation of macrophages in the adventitia is seen throughout AAA progression [30].

Furthermore, macrophages appear to actively contribute to AAA development. CCR2 and monocyte chemoattractant protein-1 (MCP-1) interactions are important for macrophage-mediated inflammatory responses, including monocyte chemotaxis. Deficiency of CCR2 in mice limits the formation of AngII- and calcium chloride-induced AAAs [31,32]. Myeloid differentiation factor 88 (MyD88), an adaptor protein central to toll-like receptor signaling, also seems to play a pivotal role in macrophage-mediated vascular inflammation as deficiency of this molecule in macrophages diminishes murine AngII-induced AAAs [33].

T- and B-lymphocytes are frequently observed in AAAs [29,34]. A functional deficiency of CD4+ CD25+ T-regulatory cells was reported in patients with AAAs, and disruption of the balance of T-helper type 1 and type 2 cell function induces AAA in mice with allografted aortas [35].

Neutrophils are also present in human, and animal model AAAs [36]. The adhesion molecule L-selectin was found to be an important mediator for neutrophil recruitment in PPE-induced AAA formation in mice [37]. Neutrophil depletion in mice with aortic perfusion of PPE leads to attenuation of AAAs [36].

Further, many cytokines and chemokines play roles in AAA development [38]. Tumor necrosis factor (TNF)-α, a landmark cytokine in many inflammatory responses, is increased in plasma from patients with AAA and in human AAA tissues [39–41]. TNF-α-converting enzyme (TACE/ADAM17), and osteoprotegerin (a secreted glycoprotein member of the TNF receptor superfamily) are enhanced in human AAAs [39,40,42]. Genetic deficiency or pharmacological inhibition of TNF-α by administration of infliximab attenuates calcium chloride-induced AAAs in mice [43].

## 3. MicroRNA Biogenesis and Function

MicroRNA (miRNAs) are a class of well-conserved, short, non-coding RNAs that have emerged as key post-transcriptional regulators of gene expression in animals and plants. miRNAs have been described to play major roles in most, if not all, biological processes by influencing stability and translation of messenger RNAs [44]. miRNA genes are transcribed by RNA polymerase II as capped and polyadenylated primary miRNA transcripts (pri-miRNA) [45]. Pri-miRNA processing occurs in two steps, catalyzed by the enzymes Drosha and Dicer in cooperation with a dsRNA binding protein, “DiGeorge syndrome critical region gene 8” (DGCR8) [46]. In the first step, the Drosha-DGCR8 complex processes pri-miRNA into a ~70-nucleotide precursor hairpin (pre-miRNA), which is then exported to the cytoplasm. Some pre-miRNAs are produced from very short introns (mirtrons) as a result of splicing and debranching, bypassing the Drosha-DGCR8 step [47]. Nuclear export of pre-miRNAs is mediated by the transport receptor exportin 5 (XPO5) [48].

In the cytoplasm Dicer matures pre-miRNA into an imperfect RNA duplex. The strand with the weakest base pairing at the 5′ terminus is loaded into the miRNA-induced silencing complex (miRISC), and is therefore considered to be biologically active [49]. While both strands of the duplex are produced in equal amounts by transcription, their accumulation into the miRISC is asymmetric [50]. Initially, the non-miRISC strand was assumed to be an inactive passenger designated the *(star)-strand. However, systemic computational analysis has demonstrated that star-strands may contain well-conserved target recognition sites, indicating functional relevance [51]. Indeed, several recent publications have reported star-strands to be biologically active, widening the potential regulatory potency of miRNA-duplexes [52–54].

After the selected strand is loaded into the miRISC, the miRNA guides the miRISC to bind to the 3′UTR of its target sequence. The seed sequence (the first two to eight nucleotides) is considered the most important for target recognition and silencing of the mRNA [55,56]. Translation of the mRNA is inhibited after association of the miRISC with its target sequence. Efficient mRNA targeting requires continuous base pairing of the seed region to the target mRNA. Furthermore, Ago(argonaute)-proteins and the glycine-tryptophan protein of 182 kDa (GW182), core components of the miRISC, are directly associated with miRNAs, and are needed for effective translational repression, mRNA destabilization, and degradation. The exact mechanisms of translational arrest by the miRNA:mRNA complex are still a matter of debate, although both initiation and elongation steps of translation are thought to be affected [57,58].

## 4. miRs in AAA Disease

**Figure 1:**
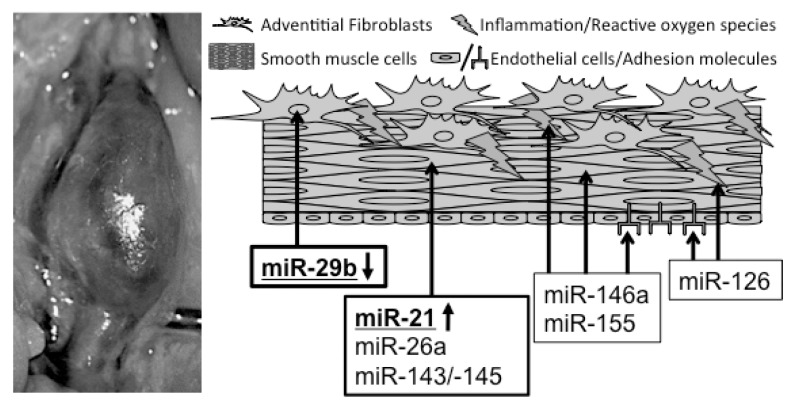
Association between microRNAs and murine abdominal aortic aneurysm formation. microRNAs (miRs) in bold and underlined have been established as regulators of aneurysm disease, utilizing gain- and loss-of function studies. All other miRs are suspected and potential disease-related modulators.

In recent years, several miRs have been found to regulate vascular pathologies, in general, and aortic aneurysm (thoracic and abdominal) disease, in particular (Table 1). We performed a systematic published literature search on articles investigating miRNA expression and function in aortic aneurysm disease. This current review focuses mainly on miRNAs that have not only been detected as being potentially dys-regulated in human aneurysmal tissue, but have also been thoroughly studied in functional experiments, thus accessing a therapeutic strategy of beneficially altering miRNA expression to limit AAA progression (Figure 1).

### 4.1. miR-21

miR-21 is considered an onco-miRNA, with increased expression in many solid tumors, where it promotes cell proliferation, migration and anti-apoptosis [64]. Data indicate that miR-21 is also highly expressed in VSMCs, and implicate it in the regulation of SMC phenotype in vascular disorders, such as post-injury neointimal lesions [65,66].

Interestingly, miR-21 stimulation induces up-regulation of smooth-muscle restricted contractile proteins through silencing of “programmed cell death protein” (PDCD)-4 expression, a known tumor suppressor protein. These findings suggest that miR-21 could regulate both VSMC contractile function [67] and proliferation [68]. miR-21 also targets multiple members of the dedicator of cytokinesis (DOCK) superfamily and modulates the activity of ras-related C3 botulinum toxin substrate 1 (Rac1) small GTPase to regulate VSMC phenotype [69].

miR-21 regulates growth and survival of VSMCs by decreasing the expression of “phosphatase and tensin homolog” (PTEN) and inducing expression of Bcl-2, resulting in pro-proliferative and anti-apoptotic effects in a carotid injury model in rats [68]. Regarding homeostasis, miR-21 promotes VSMC differentiation in response to TGF-β1 and BMP-4 [67]. These factors were shown to stimulate the processing of miR-21 in human pulmonary artery smooth muscle cells from the pri-miR to the mature miR via SMAD proteins. Additionally, miR-21 has been shown to regulate hypoxia-induced pulmonary VSMC proliferation and migration by regulating PDCD4, Sprouty 2 (SPRY2), and peroxisome proliferator-activated receptor-α (PPARα), known for their anti-proliferative and anti-migratory effects on VSMCs [70].

Interestingly, a recent report indicates that miR-21 is induced in tissue of arteriosclerosis obliterans of the lower extremities, even with <10% stenosis, and also is induced in VSMCs in response to platelet-derived growth factor (PDGF)-BB and/or hypoxia. In this report, tropomyosin 1 (TPM1) was identified as a target gene for miR-21. TPM1 reduction leads to a reduction in cytoskeletal stability, promoting VSMC proliferation and migration [71].

Furthermore, cyclic stretch has been shown to modulate miR-21 expression at the transcriptional level via FBJ murine osteosarcoma viral oncogene homolog (c-fos/AP-1) in cultured human aortic SMCs [72]. While moderate stretch is essential for maintaining vessel wall structure and vascular homeostasis [73], exacerbated stretch, as in hypertension, could promote pathological vascular remodeling by stimulating SMC proliferation, apoptosis, and migration and abnormal extracellular matrix deposition [74,75].

In endothelial cells (ECs), prolonged shear stress up-regulates the expression of miR-21 through modulation of the phosphatidylinositol-4,5-bisphosphate 3-kinase (PI3K)/v-akt murine thymoma viral oncogene (Akt) pathway, which leads to an increase of nitric oxide (NO) production while reducing apoptosis [76]. miR-21 is also expressed in endothelial progenitor cells (EPCs), where it suppresses high mobility group AT-hook 2 (Hmga2) expression, a chromatin-associated protein that modulates transcription through altering chromatin structure. Thus, inducing overexpression of miR-21 decreases proliferation and limits EPC angiogenesis *in vitro* and *in vivo* [77].

In regards to aortic dilatation, we discovered that miR-21 was significantly up-regulated in two established murine models of AAA disease, the PPE-infusion model in C57B/L6 mice and the AngII-infusion in ApoE^−/−^ mice [59]. Out of the aforementioned VSMC-specific miR-21 target genes that alter proliferation and apoptosis, PTEN was the only target gene to be significantly down-regulated at three different time points during aneurysm development and progression. PTEN, a lipid and protein phosphatase and important tumor suppressor gene, acts as a key negative regulator of the PI3K pathway. Systemic injection of a locked-nucleic-acid (LNA) modified antagomiR against miR-21 diminished the pro-proliferative impact of down-regulated PTEN, leading to a significant increase in expansion of AAAs. Further down-regulation of aortic PTEN with a pre-miR-21-loaded lentivirus had significant protective effects on aneurysm expansion by inducing massive proliferation in the aortic wall in both murine models [59].

As mentioned above, smoking is considered to be the major modifiable risk factor for AAA disease. In our study*,* nicotine (a major constituent of tobacco smoke) accelerated AAA growth in both murine aneurysm models, and caused an augmented increase in miR-21 levels, which appeared to be a protective response to limit further aneurysm expansion and rupture. *In vitro* studies utilizing human aortic SMCs and ECs, as well as adventitial fibroblasts showed aortic SMCs to be the most responsive to miR-21 modulation. Our group also showed that miR-21 induction in nicotine, as well as AngII and interleukin-6 (IL-6) pre-treated SMCs, is dependent on NF-κB signaling. In support of these findings, we found increased expression of miR-21 and down-regulated PTEN in samples obtained from human AAA patients undergoing surgical repair of their enlarged infrarenal aorta compared to control abdominal aorta from organ donors. Notably, miR-21 was even further up-regulated (with PTEN being further decreased) in smokers with AAA disease compared with non-smokers [59].

### 4.2. miR-26a

Employing *in vitro* experiments with human aortic SMCs, Leeper and colleagues [60] found that miR-26a promotes the synthetic phenotype through regulation of SMAD1 and SMAD4, contributing to the regulatory circuit of TGF-β signaling-associated pathways. Overexpression of SMAD-1 and SMAD-4 was inducible with anti-miR-26a treatment. In two mouse models of aneurysm formation (PPE- and AngII-infusion), miR-26 levels were decreased, which might contribute to AAA formation through enhanced SMC apoptosis. Thus, miR-26 regulation in aneurysmal tissue with AAA development may in fact be causal, and not compensatory.

### 4.3. miR-29b

The miR-29 family of miRs contains three members (miR-29a, miR-29b, and miR-29c) that are encoded by two separate loci, giving rise to bi-cistronic precursor miRs (miR-29a/b1 and miR-29b2/c). This family targets numerous gene transcripts that encode ECM proteins involved in fibrotic responses, including several collagen isoforms (e.g., COL1A1, COL1A2, COL3A1), fibrillin-1, and elastin (ELN) [78], and is known to modulate gene expression during development and aging of the aorta [61] and during the progression of aortic aneurysms [61,62].

Other fibrosis-related responses and diseases, such as liver [79] and kidney fibrosis [80], systemic sclerosis [81], as well as cardiac fibrosis in response to myocardial ischemia [78], have all been linked to repressed levels of miR-29. TGF-β-associated pathways are important regulators of miR-29 expression, leading to triggering of the fibrotic response by decreasing miR-29 levels in cardiac fibroblasts, hepatic stellate cells, and dermal fibroblasts, and leading to a substantial increase in the aforementioned ECM target genes [78,81,82].

Based on these observations, miR-29 seems to be a crucial regulator of aortic aneurysm disease through modulating genes and pathways which are responsible for ECM composition and dynamics. We found that miR-29b was the only member of the miR-29 family to be significantly down-regulated at three different time points during murine AAA development and progression [82]. Further decreasing of miR-29b expression with a LNA-anti-miR-29b led to an acceleration of collagen encoding gene expression (COL1A1, COL2A1, COL3A1, COL5A1), as well as elastin (ELN). Furthermore, matrix-metalloproteinases-2 and -9 (MMP2 and MMP9) were down-regulated in LNA-anti-miR-29b-transduced mice. These results were reproducible in two independent mouse AAA models, (PPE-and AngII-infusion), and led to a significant decrease in aneurysm expansion compared to a scrambled-control-miR injected group.

Human AAA tissue samples displayed a similar pattern of reduced miR-29b expression with increased collagen gene expression in comparison to non-aneurysmal organ donor controls. These results suggest that the aortic wall, which weakens due to steadily increasing diameter, acts to induce expression of collagens by repressing miR-29b levels, providing additional support to the aortic wall in an attempt to limit the risk for rupture.

Aging is a well-established risk factor for aneurysm development. Boon *et al.* were the first to publish a study connecting miR regulation to aortic dilatation and aging. They discovered that expression of the miR-29 family was increased in the aging mouse aorta [61]. Rather than utilizing the more commonly employed ApoE^−/−^ or LDL receptor^−/−^ mice, Boon and colleagues studied AngII infusion in 18-month-old C57BL/6 (wild type) mice. In these mice, AngII infusion increased miR-29b expression in samples derived from the entire aorta, which would seem to suggest that with aging the protective role of miR-29b during AAA development may be diminished. In accordance with our aforementioned results, Boon *et al.* found that systemic treatment with an LNA-modified anti-miR-29b significantly increased the expression of collagen isoforms (COL1A1, COL3A1), as well as ELN, and decreased suprarenal aortic dilatation in aged AngII-treated mice.

## 5. miR-143/145

Probably the most extensively studied miR in VSMC pathology is the miR-143/145 cluster, which is transcribed as a bi-cistronic transcript from a common promoter, which in turn is regulated by serum response factor (SRF), myocardin, and myocardin-related transcription factor-A [83]. MiR-143/145 is dramatically reduced in several vascular disease models, e.g., carotid balloon/wire injury, carotid ligation in rats, and in ApoE^−/−^ mice [83–85].

miR-143/145 alters SMC phenotypic switching in response to vascular injury, influencing both the synthetic/proliferative and the contractile/differentiated states [63,83–86]. Studies from several different groups have shown that these effects are partly mediated by targeting of multiple transcription factors, including KLF4, KLF5, and ELK-1 [84–86]. Further, down-regulation of miR-143/145 is sufficient to up-regulate PDGF receptor (PDGF-R), protein kinase C (PKC) epsilon, and fascin, an actin bundling protein of podosomes. These last are thought to be necessary for vascular wall matrix remodeling, potentially affecting the progression of aortic dilatation [87]. Interestingly, one of the first reports regarding the role of miRs in aneurysm disease showed that miR-143/145 expression is reduced in aortas from patients with thoracic aortic aneurysm, permitting dedifferentiation of aortic VSMC with a resultant decrease in contractile function [63].

Finally, miR-143/145 may be secreted in microvesicles derived from ECs (which otherwise do not usually express these miRs) [84]. It has been proposed that shear stress-induced KLF-2 may stimulate expression of miR-143/145 in ECs [88], leading to miR secretion in microvesicles and transfer into VSMCs [84]. EC-derived microvesicles containing miR-143/145 can reduce atherosclerotic lesions when injected into ApoE^−/−^ mice [88].

## 6. Other miRs

A growing body of literature highlights the role of miRs in the regulation of angiogenesis and inflammation [12–14,89]. Smooth muscle degradation, along with decreased VSMC proliferation, decreased ECM synthesis and impaired ECM remodeling, have all previously been linked to AAA development. Clearly, these contributing mechanisms of aortic dilation may be regulated through miRs. However, the miRs described below have not yet been directly tied to aortic aneurysm initiation, propagation, or rupture.

### 6.1. miR-126

One of the most intriguing miRs as regards vascular inflammation is miR-126, an EC-enriched miR, which negatively regulates VCAM-1 expression [90,91]. Apoptotic bodies are released from ECs during atherosclerotic progression, and have been shown to contain miR-126. miR-126 decreases the expression of G-protein signaling 16 (RGS16) in ECs, thereby up-regulating the chemokine (C-X-C motif) ligand 12 (CXCL12) receptor. CXCL12 activation then decreases EC apoptosis and recruits progenitor cells at the lesion site, reducing the atherosclerotic burden *in vivo*, and contributing to plaque stabilization [92].

### 6.2. miR-146a

Alterations associated with aging in blood vessels include a decrease in compliance and an increase in vascular inflammatory response, which could promote AAA propagation. Several reports show dysregulation of miRs in the vasculature during aging. In particular, miR-146a expression is decreased in senescent ECs. It targets NADPH oxidase 4 (NOX4), decreasing reactive oxygen species (ROS) production. These data suggest that the reduction in miR-146 expression potentially enhances aging effects through NOX4-derived ROS [93]. In another study, miR-146a and KLF4 were found to form a feedback loop, regulating each other’s expression and VSMC proliferation. The authors propose that miR-146a regulates KLF4, which competes with KLF5 binding to the miR-146a promoter to inhibit transcription [94].

### 6.3. miR-155

miR-155 is another miR of potential interest in AAA disease progression due to its effects on the renin-angiotensin-system (RAS). miR-155 is induced by TNF (which independently has been shown to contribute to AAA development) [95], and then negatively regulates the expression of the transcription factor “v-ets erythroblastosis virus E26 oncogene homolog 1” (Ets-1) [96]. AngII-induced overexpression of miR-155 results in a decrease in Ets-1, affecting expression of downstream targets such as VCAM-1, fms-related tyrosine kinase 1 (FLT1) and MCP1, and impairing lymphocyte adhesion to ECs [96]. miR-155 also has been shown to target the angiotensin II type 1 receptor (AT1R), resulting in decreased AngII-induced migration of ECs [96].

While the above-described effects of miR-155 might suggest an anti-inflammatory role, Nazari-Jahantigh *et al.* validated miR-155 in macrophages as a crucial component of atherosclerosis development. In these cells, miR-155 promoted the expression of MCP-1/CCL2, and directly suppressed Bcl-6, a transcription factor that inhibits NF-κB [97]. It has also been described that hematopoietic deficiency of miR-155 increases atherosclerotic plaque size and instability [98], possibly by inhibition of lipid uptake and inflammatory responses in monocytes. Clearly, findings thus far regarding the role of miR-155 have been somewhat ambiguous.

In addition to these miRs, Pahl *et al.* examined miR-regulation in human abdominal aortic tissue of patients undergoing elective open repair with samples collected at autopsy or obtained from a pre-existing tissue biobank [99], utilizing microRNA-array. Out of a total of 847 miRs, 3 miRs presented as significantly up- (miR-181a*, miR-146a, miR-21) and 5 miRs as down-regulated (miR-133b, miR133a, miR331-3p, miR30c-2*, miR-204) in patients with AAAs compared to controls. However, using an additional tissue set, qRT-PCR was only able to confirm the down-regulated miRs from the array.

## 7. Therapeutic Approaches Using miR Modulators

The identification of both the underlying causes of vascular disease, as well as appropriate interventions, remain great challenges to both basic vascular biology and everyday clinical practice. The traditional methods of drug design, involving enzymes, cell surface receptors, and other proteins, appear sometimes less effective in the treatment of cardiovascular diseases, due to the highly sensitive nature of the targeted systems.

In this dismaying scenario, the discovery of an entirely new method of gene regulation by miRs, and their recent validation as markers and modulators of vascular functionality during pathological conditions, provide new hope for innovative therapies. Research in recent years has recognized the crucial regulatory roles that miRs play in vascular diseases such as myocardial infarction, stroke, and aortic aneurysm [100].

Intriguingly, miRs also appear to represent valid therapeutic targets, because modulation of their expression *in vivo* with either antisense RNA molecules or miR-mimics/pre-miRs has been shown to effectively modulate cardiovascular disease in various animal models [101]. Inhibition or overexpression of a single miR can induce or attenuate pathological responses in the cardiovascular system, as a result of the regulated coordination of numerous target genes involved in complex physiological and disease phenotypes. The most important difference between modulating miRs, and the traditional therapeutic approach is that standard drugs typically interact with specific cellular targets, whereas miRs have the capability of modulating entire functional networks [102].

miR modulation is performed by supplying antagomiRs (or anti-miRs; synthetic reverse compliments of oligonucleotides) that bind to a target miR and silence it, or by using pre-miRs/miR-mimics that act similarly to the original miR [101]. Recent animal and even human efficacy data indicate that antagomiRs have the potential to become a whole new class of drugs. These inhibitors of miR expression have several significant advantages, which make them very attractive from a drug development standpoint, including small size, as well as frequent conservation of their target miRs across species. Using lessons learned from antisense technologies (e.g., siRNA), potent oligonucleotide chemistries to inhibit miRs are currently being investigated [103]. These efforts have given rise to candidates that bind to their putative miR targets with remarkable affinity and specificity, and which have desirable drug-like qualities, including increased stability and favorable pharmacokinetics.

The most common type of modification being utilized to protect antagomiRs from immediate degradation *in vivo* is the addition of a locked nucleic acid (LNA). LNA contains a class of bicyclic RNA analogs in which the furanose ring in the sugar-phosphate backbone is chemically locked in a RNA-mimicking N-type (C3′-endo) conformation by the introduction of a 2′-*O*,4′-*C*-methylene bridge. This modification leads to nuclease resistance, as well as an increase in binding affinity to the targeted miR, which is accomplished by Watson-Crick complementary base pairing [104]. Regarding the use of antagomiRs in humans, there have been no immunogenic or toxicological safety issues reported to date. However, the major drawback of these substances at this point seems to be the necessity of repeated delivery of doses for long-term therapeutic effects. This becomes a critical issue when the route of delivery is an invasive procedure, such as systemic injection [105]. The antagomiR that has advanced the farthest in clinical trials to date is Miravirsen (anti-122) for patients with chronic hepatitis-C (HCV) infections. Recently published data from a Phase 2a trial demonstrated that the drug was not only safe, but also well tolerated, providing prolonged antiviral activity well after the last dose of monotherapy [106].

Unlike antagomiRs, the prospect of delivery of injectable, naked miR-mimics and/or pre-miRs has remained problematic. For now, lenti- as well as adeno-associated viruses (AAV) represent efficacious delivery platforms for miRs, but these carry the risks common to most gene therapies. Lentiviral vectors, for example, are derived from HIV type 1 (HIV-1), and thus the production of wild-type HIV through homologous recombination of the virus remains a major safety concern. However, recent lentiviral vector developments permitting deletion of the U3 promoter region of the long terminal repeats from the virus, leading to self-inactivation, may resolve this issue, making them a promising vector for future applications [107].

miR-mimic and pre-miR development also present difficulties related to the need to deliver synthetic RNA duplexes in which one strand (the “guide” strand) is identical to the miR of interest, while the complementary strand (“passenger” strand) is modified to increase stability as well as cellular uptake. Apart from the problems involved in permitting cellular uptake of double-stranded miR-mimics, the passenger strand has the potential to counter-productively act as an antagomiR [105]. Given the above limitations, the development of miR mimics, which do not require a viral vector represents an important therapeutic goal. Some preclinical studies have achieved this in murine models by packaging synthetic miR duplexes within lipid nanoparticles [108,109].

In summary, the ability to modulate miR activity through systemic delivery of miR inhibitors or mimics without toxicity provides unprecedented opportunities for intervening in disease processes. While challenges such as potential off-target effects and the urgent need for local and/or cell-type specific delivery mechanisms remain, the pace of discovery in this field portends new, feasible clinical therapeutic approaches in patients.

## 8. miRs as Biomarkers in AAA Disease

At the outset, it is necessary to point out that, to date, no easily accessible and reproducibly measurable biomarker has been identified with prognostic value for AAA growth, or even for the potential to rupture [1,110].

Recently, miRs have received much attention regarding their suitability as biomarkers for vascular disease. Following pioneering work from the cancer field, several cardiovascular studies have found substantial variations in miR expression in numerous clinical specimen subtypes (e.g., blood, urine, saliva, *etc*.) [111–114]. Measuring levels of circulating miRs has several advantages and offers novel opportunities. For example, as with nucleic acids, miRs can be both amplified and detected with high sensitivity and specificity. Also, miR-microarrays and quantitative PCR (qPCR) methodology allows the quantification of many miRs in a single experiment. There is evidence that the combined analysis of many miRs and their co-expression patterns (miR networks) enhances their predictive power as biomarkers. Furthermore, miRs are relatively stable over time in human blood and appear to be protected from degradation through various mechanisms [115].

Despite this, the quantitative analysis of miRs in material such as blood and urine comes with certain disadvantages. Firstly, the concentrations of most circulating miRs are typically very low (with the exception of whole blood samples), making reliable quantitation and normalization a challenge with existing technology. Also, there exists no consensus for miR normalization controls. Beyond this, current qPCR and microarray technologies are still quite time-consuming (several hours) compared with some protein-based biomarker tests such as troponin or C-reactive protein, which can offer results within minutes [116]. For now, the added value of miR-based biomarkers remains to be established by more rigorous testing and optimization.

Despite these hurdles, several laboratories have already obtained profiles of circulating miRs in cardiovascular disease and explored their biomarker potential. Immediately apparent are certain inconsistencies between studies, where the same or highly similar settings have been studied. This is partially attributed to the current immaturity of the field, which still includes technical issues such as variability of RNA extraction protocols, different means of nucleic acid detection, and the aforementioned normalization procedures. However, many studies are also simply clinically underpowered, and/or do not use appropriate controls matched for potentially confounding factors such as age, sex, medication, comorbidities, and tissue source. Also, there has been minimal comparison of miRs to traditional reference biomarkers.

The first study to look at expression levels of circulating miRs in AAA disease was performed by Kin *et al*. The authors investigated a subset of miRs, which they identified to be significantly altered in abdominal aortic tissue samples from patients with AAA undergoing surgical repair when compared with non-aneurysmal thoracic aortic specimen from patients undergoing aortic valve replacement [117]. Interestingly, miRs that were up-regulated in AAA tissue samples appeared significantly down-regulated in plasma from patients with AAA compared to a small group of healthy volunteers. These included miRs-15a/b, -29b, -124a, -126, -146a, -155, and -223. Clearly, further studies in larger cohorts are necessary to explore the diagnostic, and, even more important, the predictive capabilities of miRs as biomarkers in AAA disease.

## 9. Summary and Perspectives

The demonstration that miRs play crucial roles in cardiovascular disease and can be easily regulated *in vitro* and *in vivo* by antagomiRs and pre-miRs/miR-mimics has tremendously accelerated miR research and nourished hopes that the agents used and verified in animal models could some day be employed in humans with AAA disease. miRs represent a relatively young, but rapidly advancing, field of basic biological and translational research with potentially new and innovative therapeutic applications. For vascular diseases in particular, the availability of local (coated stents and/or balloons) or cell type-specific delivery mechanisms would significantly increase the value of miR therapeutics in everyday clinical practice.

## Figures and Tables

**Table 1:** Regulatory role of microRNAs in murine abdominal aortic aneurysm (AAA) disease models (AFB = adventitial fibroblasts; AngII = angiotensin II; ASMC = aortic smooth muscle cells; PPE = porcine pancreatic elastase).

microRNA	Model of AAA induction	Effect on AAA progression
miR-21	PPE-infusion in C57BL/6 mice and AngII-infusion in *ApoE*^−/−^ mice [59]	Regulates proliferation and apoptosis in ASMCs via PTEN/PI3K/AKT; induction of miR-21 through NFκB
miR-26a	PPE-infusion in C57BL/6 mice and AngII-infusion in *ApoE*^−/−^ mice [60]	Inhibition of ASMC-differentiation via SMAD-1 and SMAD-4 depression
miR-29b	AngII in 1.5-year-old C57BL/6 [61]; PPE-infusion in C57BL/6 mice and AngII in *ApoE*^−/−^ mice [62]	Modulating the fibrotic response in aortic wall through several collagen isoforms; repression of miR-29b in AFBs through TGF-β
miR-143/145	miR-143/145 knockout and *ApoE*^−/−^ mice [63]	Regulation of ASMC homeostasis and differentiation
